# Somatic intronic microsatellite loci differentiate glioblastoma from lower-grade gliomas

**DOI:** 10.18632/oncotarget.2076

**Published:** 2014-06-05

**Authors:** Enusha Karunasena, Lauren J. McIver, Brian R. Rood, Xiaowei Wu, Hongxiao Zhu, Jasmin H. Bavarva, Harold R. Garner

**Affiliations:** ^1^ Virginia Bioinformatics Institute, Medical Informatics and Systems Division; Blacksburg, VA; ^2^ Center for Cancer and Blood Disorders at Children's National Medical Center; Washington, D.C; ^3^ Department of Statistics at Virginia Tech; Blacksburg, VA

**Keywords:** GBM, microsatellite, glioma, oligodendroglioma, helicase, ubiquitin proteasome system

## Abstract

Genomic studies of glioma sub-types have amassed new disease specific mutations, yet these only partially explain how mutations are linked to predisposition or progression. We hypothesized that microsatellite variation could expand the understanding of glioma etiology. Furthermore, germline markers for gliomas are typically undetectable; therefore we also hypothesize that the predictability of cancer-associated microsatellite loci in germline DNA may support the current hypothesis of a glioma cell of origin.

In this study, “normal” germline exome sequenced DNA from the 1000 Genomes Project (n=390) were compared with exome sequences from germlines of subjects with WHO grade II and III lower-grade glioma (LGG, n=136) and WHO grade IV glioblastoma (GBM, n=252) from The Cancer Genome Atlas to identify microsatellite loci non-randomly associated with glioma. From germline data, we identified 48 GBM-specific loci, 42 Lower-grade glioma specific loci and 29 loci that distinguish GBM from LGG (p≤ 0.01). We then attempted to distinguish WHO grade II glioma (n=67) from GBM resulting in 8 informative loci. Significantly, in all glioma grades, comparisons between tumor and matched germline sequences demonstrated no significant differences in these variants (p≥ 0.01). Therefore, these microsatellite loci are considered to be components of grade-specific signatures for glioma which distinguish germline sequences of individuals with cancer from those of individuals that are “normal”. In order to better understand the significance of these loci, we identified biological processes enriched in genes with these variants. Most strikingly, six helicase genes were enriched in the GBM cohort (p≤ 1.0 ×10^−3^). The preservation of these glioma-specific loci could therefore serve as valuable diagnostic and therapeutic markers; especially since the heterogeneity of tumor cell populations can obscure the identification of mutations preceding a metastatic phenotype.

## INTRODUCTION

Every year in the U.S. approximately 10,000 new adult malignant brain tumors are diagnosed[1] with the most common being glioblastoma (GBM; WHO grade IV astrocytoma)[2]. Treatment with radiation and chemotherapy yields a median survival of only 11-14 months; therefore, GBM is considered a terminal diagnosis [3]. There are three main histological groups of adult gliomas: astrocytoma (A); oligodendroglioma (OD) which are slower-growing but rarely progress to GBM; and mixed glioma such as oligoastrocytomas (OA), a mix of A and OD[4, 5]. Astrocytoma is graded from I to IV according to the World Health Organization's (WHO) classification criteria while OD and OA are primarily in grades II and III. Lower grade adult astrocytomas can progress to higher grade tumors. Recurrence after therapy is common with A, OA, and most OD and is generally associated with more aggressive and infiltrative tumors [6].

High-throughput sequencing studies of tumor genomes have produced new molecular markers that have enhanced classification of GBM and highlighted molecular pathways that propagate pathogenesis and progression [6-9]. In this study, we have chosen to identify and characterize a specific component of glioma genomics- DNA microsatellite variants – a feature that is understudied relative to SNPs or epigenetic markers. Microsatellites (MST) are short tandem repeated sequences with variable lengths, making them excellent markers of disease[10]. Repetitive nucleotide sequences account for 55% of the intergenic region of the genome with 3% of these considered microsatellite repeats[10, 11]. Microsatellites can be found in both coding and non-coding regions, are generally 10-15 nucleotides in length and are found as monomers, di-, tri-, penta-, or hexamers[11, 12]. Expansion of tri-nucleotide repeat sequences are notably contributive to over 40 different neurological disorders including Fragile X, Huntington's and Parkinson's Disease[13]. These alterations are hypothesized to lead to changes in gene regulation through modified binding sites for transcription factors, splicing machinery, or microRNAs [10, 12, 13]. Thus, changes to microsatellite sequences elicit phenotypes and contribute to diseases, including cancer.

Therefore, we hypothesize that gliomagenesis is correlated with microsatellite variability at specific loci in germline DNA. Additionally, we demonstrate that germline (non-tumor) DNA is representative of the tumor and these glioma-specific microsatellite loci discriminate glioma grades (specifically II versus IV). With validation, such markers could be developed to aid clinical predictions. We further hypothesize that these loci could influence tumor biology, including a potential contribution to early glioma tumorigenesis.

## RESULTS

### The two populations studied are GBM and lower-grade gliomas (LGG)

Classifications were provided by The Cancer Genome Atlas (TCGA) and we have chosen to discuss our results using the same assemblage for scientific comparisons (as such, lower-grade gliomas are a mix of glioma types that are non-GBM samples). Microsatellite loci were identified from exome sequencing data for a cancer germline and a “normal” population. Those sequences that varied in length significantly between the two populations were compiled to create a ‘signature’ list (see Table 1). Signatures consist of variant allelic pairs (genotypes) from cancer-associated microsatellite loci (CAMLs). Three signatures are reported: GBM vs normals, LGG vs normals, and GBM vs LGG.

**Table 1:** Genomic Microsatellite Loci Identified from GBM, LGG, and Grade II Germline Sequences The number of informative loci that passed all statistical tests and differentiated CAMLs from “normal” genomes included 48 GBM loci and 42 LGG loci; of these, 10 of the signature loci in GBM overlapped with those in the LGG signature. Eight loci from 67 Grade II germline samples were uniquely identified when compared with GBM (average detectable loci in GBM 4.3 loci (± 1.6)).

	Total MST Loci (Exome Sequences)	Significant MST Genotypes (p≤ 0.01)	FDR Corrected CAML Genotypes (Signature)	Average Detectable Signature Genotypes per Sample
GBM	26,021 (SD ± 4,859)	179	48	13.1 (±6.6)
LGG	26,596 (SD ± 2,392)	146	42	15.1 (±5.9)
Grade II	26,427 (SD ± 2,333)	75	8	4.2 (±1.7)

### Gliomas Compared with Normal Sample Sequences:

**FIGURE 1:**
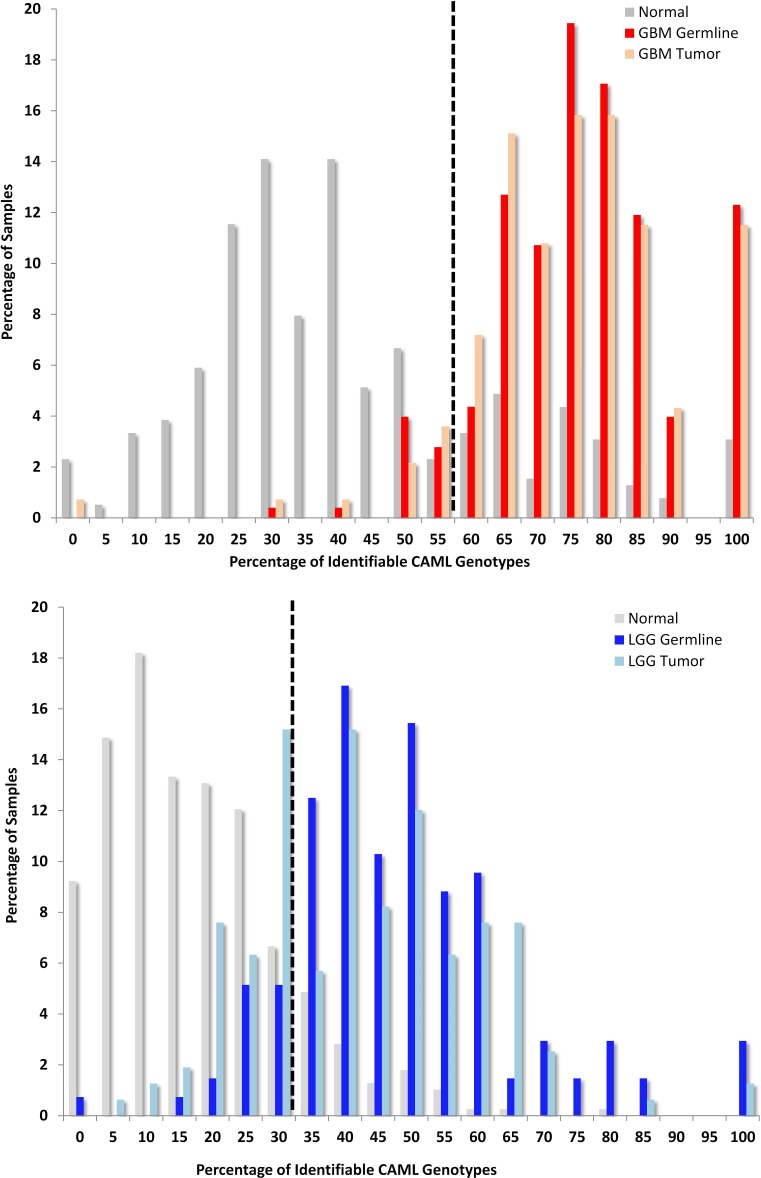
Variant Glioma Microsatellite Loci Significantly Differ from Normal Germlines The y-axis describes the percentage of samples and the x-axis shows a given fraction of detectable CAML genotypes; a receiver operator characteristic (ROC, see supplement) was used to determine the cut-off thresholds, which are 57% GBM and 35% LGG; illustrated by a black hashed-vertical line). (1A.) GBM: First, we compared TCGA GBM to an ethnically matched “normal” population in the 1000 Genomes Project (1kGP) and calculated a sensitivity of 94% and specificity of 77% (corresponding GBM tumor sensitivity is 96% and specificity is 75%). Second, we compared LGG to 1kGP and determined the sensitivity to be 91% and specificity at 86% (LGG tumor sensitivity is 84% and specificity is 86% compared with 1kGP). (1B.) LGG: LGG encompassed WHO grade II and III A, OD, and OA. We identified the cut-off again using ROC analysis at 32%. Sixty-six percent of LGG samples contained ≥ 40 percent of LGG-specific CAML genotypes.

**FIGURE 2:**
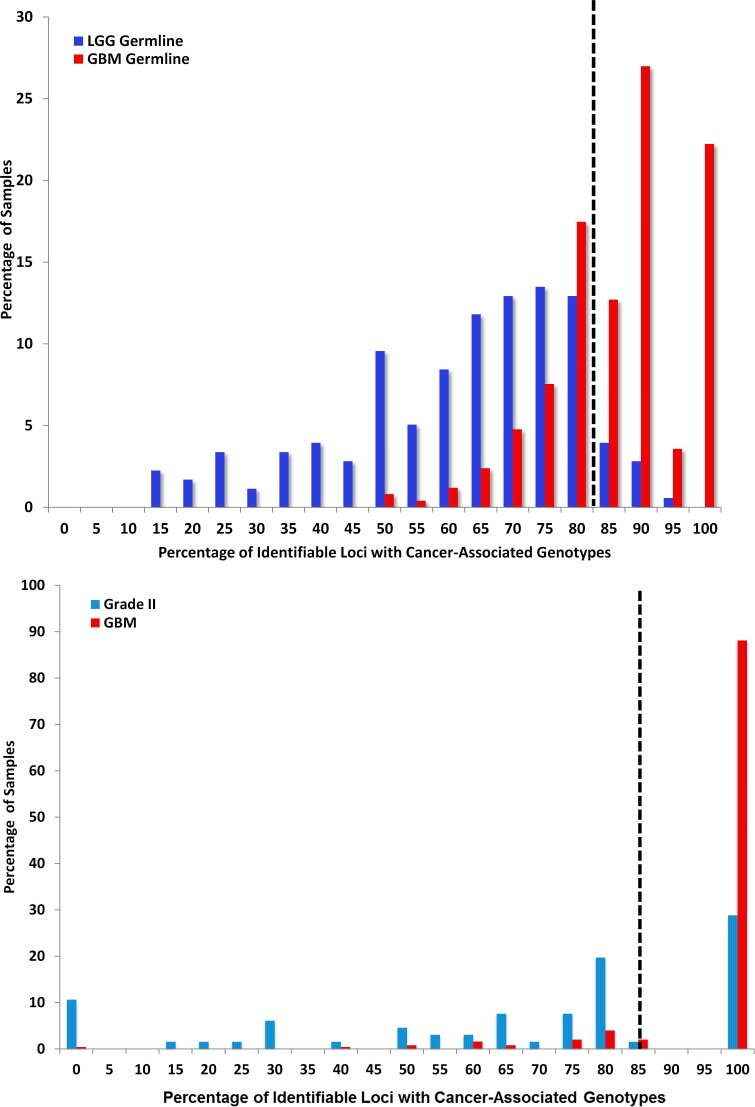
Variant Microsatellite Loci Distinguish Lower and High Grade Gliomas (2A.) LGG versus GBM: CAML genotypes shared between LGG and GBM sequences were identified. GBM germline sequences contain 90% of the signature CAMLs, whereas 3% of LGG germlines were represented in the same group (2B.) Grade II versus GBM: Seventy-percent of Grade II A, OA, and some OD progress to Grade III or IV[40]. We identified Grade II glioma signature genotypes; we then measured the percentage of samples (GBM and Grade II) with these CAMLs. In this analysis we identified eight Grade II specific genotypes. The sensitivity of these loci is 90% and specificity is 70%, with a cutoff of 85%. GBM germline sequences shared an abridged population of LGG genotypes; 82% of identifiable Grade II germline genotypes were also found in GBM samples. (2C) Below 82%, the percentage of CAML genotypes in LGG are more enriched. In Grade II samples, ≥ 75% of loci could be located in 26% of samples versus 4% of GBM. The genotypes identified in 19% of Grade II samples with 80% of CAMLs were located with the following genes (in order of significance): *KIAA1219* (13 samples), *SNX17* (12 samples), *SACMIL* (9 samples), *MYCBP2* (8 samples), *GFM1* (7 samples), *COPS4* (6 samples), and *CDC16* (1 sample). All eight signature loci were identifiable in the majority of Grade II, GBM, and the general population (1kGP; data not shown). We compared GBM with LGG germline and calculated sensitivity at 74% and specificity at 90% (tumor analysis shows sensitivity at 76% and specificity at 72%). We identified a sensitivity of 90% and specificity of 70% for Grade II loci compared with GBM.

The distribution of CAML genotypes found within samples differentiates the germlines of patients when compared to cancer-free controls. Considering the GBM signature, 19% of GBM germline samples had ≥75% of CAML genotypes and 16% of GBM tumors also had ≥75% of these loci (Fig. 1A). Additionally, 100% of the CAML genotypes from the GBM signature were discovered in 12% of GBM germline samples, while only 3% of “normal” samples demonstrated similar results. Also described in Figure 1 are normal samples compared with LGG: here the largest cluster of LGG germline samples (20%) had 50% of the detectable CAML genotypes (Fig. 1B). In an alternative analysis, we removed Grade III astrocytomas (n=28; additionally, samples with non-descript pathology (16 described as NA and 2 as UNK)) to create a new LGG signature (see Fig. S3): this signature is composed of 24 CAMLs, of which all but 4 loci were in the LGG data that are described in Fig. 2B. Importantly, all 4 of these loci were identified as significant in the analysis associated with Figure 2B. Also, given the heterogeneous population in the LGG cohort, we isolated OD Grade II and Grade III (n=59) and show the distribution of LGG genotypes in these samples compared with the total LGG germline population (n= 92); the largest population of OD (15%) had 25% of LGG CAMLs (see Fig. S3).

### Glioma Grades Compared with GBM Sequences:

We compared LGG and GBM germline sequences (Fig 2A) to determine if there was information embedded in patient germlines that forecast tumor grade and discovered 29 signature CAMLs that distinguish LGG from GBM. Eleven of these loci were in LGG, CAML genotypes (p≤ 0.01) and 10 loci were found among significant LGG microsatellite (MST) genotypes. Two loci (9:52626-52640 and 2:91886031-91886042) are in the GBM signature and one locus was found amongst significant GBM genotypes (*SSX2*). Our next goal was to determine, if specific microsatellite variants could be further used to distinguish Grade II from GBM. Most Grade II samples (75%) had between from 0% to 80% CAMLs, while 90% of GBM samples exhibited 100% CAMLs (Fig. 2B).

### Gene Ontologies & Cell Functions Important to LGG & GBM

Molecular, cellular, and biological processes significantly (p ≤ 0.1) associated with signature loci were analyzed using DAVID annotation tools[14]. From these annotations, we further evaluated individual genes and their potential roles in GBM biology, as described in detail in Tables S1, S2, and S3. Genes proximate to our CAMLs primarily contribute to RNA- processing and the ubiquitin proteasome system (UPS). Both of these cell functions are activated by interferon in viral pathogenesis, and both are important in identifying and degrading/correcting mis-folded macromolecules (e.g. – RNA, DNA, and proteins). Additionally, malfunctions by the UPS are frequently linked to neuropathies and gliomas [15].

### Regulatory Elements near CAMLs in LGG & GBM:

We discovered CpG islands (≤10 kB from MST loci) and transcription factor binding sites (TFBS, ≤ 40 bases of MST loci) near CAMLs. Ten MST loci from GBM and 9 LGG loci (with 3 loci shared between both glioma cohorts) were linked to CpG islands. Nine MST loci from GBM and 20 MST loci in LGG (with 2 loci shared between both populations) were located within or near TFBS (see Table S1). Illustrated in Figure S2 are the six helicase genes with CAMLs from the GBM signature: all six loci are within ENCODE methylation marker sites (H3KMe1 and H3KMe3), 5 of the loci are within transcription factor binding sites, and 2 are located within expressed sequence tags (EST).

### Correlation between CAMLs & Gene Expression:

We measured changes in gene expression and differences in transcripts for those genes with CAMLs, and RNA transcripts from glioma tumors (LGG and GBM) were compared with normal adult brain RNASeq data, previously described by Ameur, A. et. al, (Table 2) [16]. In our analysis from tumor versus “normal” germline samples, expression data demonstrated significant (p≤ 2.0-fold) down-regulation of *LNX2* and *FGD6* and up-regulation of *CRISP1* in LGG. In GBM, a significant down-regulation of *SEMA3E* and up-regulation of *SLC44A4* were measured (see Tables S1 and S2).

**Table 2:** GBM and LGG Genes with CAMLs and Novel Transcript Isoforms RNASeq data for GBM and LGG were screened for transcript diversity and abundance these were compared with normal adult brain transcript data, previously analyzed[16]. For GBM and LGG, described are genes for which an isoform was the most abundant transcript; reported are the average copies for these transcripts and the average copies of total transcripts for a gene. We also identified genes in which transcripts were exclusively identified in GBM and/or LGG compared with normal brain tissue. Ten CAMLs were shared between GBM and LGG, of these we identified three that were expressed in both sample populations; in both GBM and LGG the most abundant transcript for FUBP3 was an isoform compared with normal tissue which had one transcript that was a conserved sequence. RNASeq data from the Illumina BodyMap 2.0 Project was used to determine if transcripts had been identified for those genes which only showed expression in GBM or LGG in normal brain, all genes demonstrated positive expression in these data.

GBM			
Gene	Transcript Diversity	Avg. Copy of Gene	Avg. Copy of Novel Isoform
DICER1 (GBM)	16	0.15	1.2
DICER1 (N)	4	5.5	
FGFR2 (GBM)	11	0.24	1.6
FGFR2 (N)	4	3	
FUBP3 (GBM)	5	0.41	0.99
FUBP3 (N)	1		
HYDIN (GBM)	17	0.48	3.45
HYDIN (N)	1		
ICA1L (GBM)	10	2.4	0.41
ICA1L (N)	3		
OFD1 (GBM)	14	0.18	0.86
OFD1 (N)	1		
POLQ (GBM)	7	0.49	1.8
POLQ (N)	1		
SPOPL (GBM)	7	1.04	0.24
SPOPL (N)	2		

EXPRESSED *ONLY* IN GBM				
Gene	Transcript Diversity	Avg. Copy of Gene	Avg. Copy of Novel Isoform	Avg. Copy of Common Transcript
TTF2	10	0.29	1.03	
ACRC	5	0.39	0.76	0.86
COL9A1	10	0.17	0.59	
CORIN	6	0.09	0.02	0.92
FRMD7	1	5		
GTPBP8	4	0.25	0.03	0.94
NSUN5	6	0.21	0.31	0.62
NUFIP1	4	0.29	0.09	0.98
RYR1	8	0.68	2.3	
SLC44A4	2	0.05	0.03	0.07
STRC	8	0.03	0.05 0.005 0.02	0.07

LGG			
CDC16 (LGG)	13	0.23	1.2
CDC16(N)	5	1.2	
RBM5 (LGG)	14	0.3	1.3
RBM5 (N)	5	2.6	

EXPRESSED *ONLY* IN LGG				
CDH16	1	0.08		
CDRT1	1	0.41		
DENND3	13	0.64	065	2.2	
LNX2	2	0.2	0.02	0.39
GUSB	8	0.35	1.3	
ARSK	5	0.1	0.01	0.38

EXPRESSED IN GBM & LGG				
EVC (GBM)	8	0.24	0.76	
EVC (LGG)	6	0.24	0.85	0.1
EVC (N)	0			
FUBP3 (GBM)	6	0.41	0.99	
FUBP3 (LGG)	5	0.36	0.86	
FUBP3 (N)	1	2		2
PSME3 (GBM)	6	0.29	0.24	0.93
PSME3 (LGG)	11	0.15	0.09	0.77
PSME3 (N)	1	3		

### Correlation between CAMLs & Glioma Associated Driver Mutations

**Figure 3:**
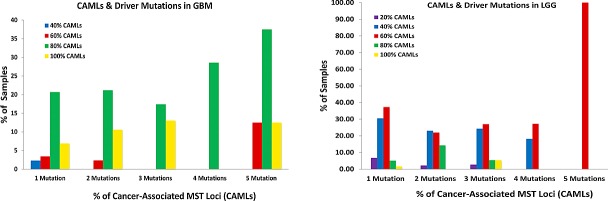

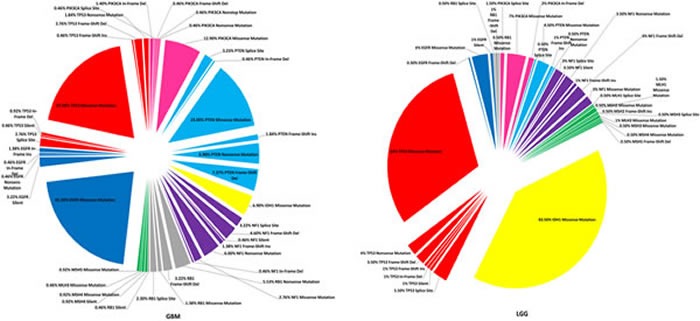

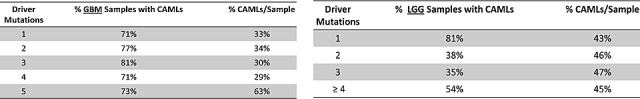
A-C Differences in Cancer-Associated Microsatellite Loci and Driver Mutations in GBM & LGG. Frequently identified somatic mutations were quantitated from DNA sequencing samples from GBM (n= 216) and LGG (n=200). Mutations in TP53, EGFR, RB1, NF1, PTEN, PIK3CA, IDH-1, and six mis-match repair genes were classified from sequencing samples and reported as a percentage of the total population for each glioma cohort. 3A: The percentage of samples with CAMLs (ranging from 20%-100%) and with up to 5 driver mutations (the maximum) are shown. 3B: A pie-chart was created to describe the distribution of mutations in those genes for GBM and LGG; mutations in p53, EGFR, and PTEN were frequently identified from GBM, while missense mutations in IDH-1 were the most common in LGG samples. Mutations in mis-match repair genes were infrequently identified from both GBM and LGG, regardless of the percentage of CAMLs identified in GBM or LGG. 3C: Samples with a given population of mutations (on average between 1-5) were examined to determine the percentage of GBM or LGG samples with CAMLs, and of these the percentage of CAMLs per sample. LGG samples contain more CAMLs compared with GBM, however, more GBM samples with CAMLs were identifiable.

We analyzed TCGA gene mutation data (i.e. INDEL, frame shift, non-frame shift etc…) to identify connections between driver mutations loci (36 mutations in total identified in 7 genes and 6 mis-match repair genes (MMR)) closely identified with gliomas and the signature. Our data show that GBM samples contain 80% of the GBM CAML genotypes regardless of the number of driver mutations found within those samples and that the majority of LGG samples contain 60% of LGG CAML genotypes. However, an increase in CAMLs was identified in most samples with an average of 5 driver mutations, in both GBM and LGG. Interestingly, LGG samples with only 1 driver gene mutation (frequently, IDH-1) were the most likely population to demonstrate CAMLs, and on average 81% of these LGG samples had 43% of the LGG CAMLs (Fig. 3C). In general both GBM and LGG populations carry similar percentages of CAMLs, regardless of the number of driver mutations.

**Figure 4:**
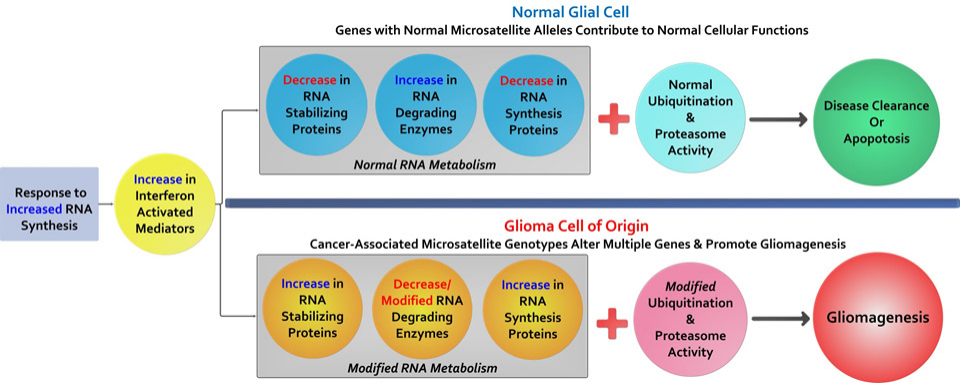
The potential contributions of cancer-associated microsatellite variants to gliomagenesis: Briefly outlined is a model to explain MST driven gliomagenesis We hypothesize that these cancer-associated variants are a component of a glioma initiating cell population enriched for CAMLs that predict functional associations with RNA synthesis, RNA degradation, and protein degradation important to transcription complexes (spliceosomes, snRNPs, snoRNPs, and snRNA). As evidenced through our data, several helicases and E3 ubiquitin ligases that are transcriptionally up-regulated by interferon signaling harbor CAMLs, suggesting that a mosaic of genes with functions ascribed to known glioma-specific biology, may work differently in glioma cells with specific and variant microsatellite genotypes. These subtle yet compounding variants may produce gene-products which further intensify genomic instability causing greater genomic alterations (gene mutations, hypermethylation, chromosome loss/breakage, etc.) favoring cancer.

## DISCUSSION

These data show microsatellite genotypes can differentiate cancer from non-cancer populations and also lower grade from GBM. Since these variant loci are identifiable in somatic DNA and are conserved in tumors lends support to the hypothesis that glioma initiating cell populations exist and are inherent to the individual and their disease.

Interestingly, we observe that a small number ≤3% of normal subjects have 100% of the GBM, CAML genotypes. This may be due to sample population age biases such that some individuals are as yet undiagnosed, or that our measure over-predicts given that the rate of gliomas in the general population is less than 3%. Therefore risk screening may be valuable only in a subset of the population presenting with an abnormal MRI. Additionally, when we inspected samples with driver mutations, observed in gliomas, the relative percentage of CAMLs is similar in individuals with 1-4 mutations. However, for individuals with 5 mutations there appears to be an increase in identifiable CAMLs which may correspond to overall instability in the genome's of these individuals. The mix of glioma types in the LGG cohort may lend to smaller populations of LGG DNA sequences with identifiable CAMLs compared with GBM (as observed in Fig. 2B, Fig 3C, and FigS3) which suggests that distinguishable and conserved MST loci may further differentiate glioma phenotypes and may be tumor specific markers. Circulating disease markers for tissue-specific cancers are progressively becoming more detectible; however diagnosing primary brain cancer markers outside of brain tissue remains poor[17]. Therefore, testing for these CAMLs from blood samples could provide a potential supplemental diagnostic resource to identify the most common and dangerous adult brain-specific cancers.

Since some microsatellites are associated with DNA ‘fragile sites’, locations within chromatin susceptible to constrictions or break-points that are linked to cancers and neuro-developmental disorders, we analyzed our MST loci to determine which are located in these regions, as a possible mechanism for tumor potentiation [18, 19]. *BRWD2*, found in our GBM signature, is located at a break-point on chromosome 10 and allelic deletions within 10p, 10q 25-26, and 19q 13.3-13.4 are the most common alterations in glial tumors[20, 21]. Given the location of the breaks, *BRWD2* is considered a candidate tumor suppressor; through our analysis we identified 80% of GBM patients versus 50% of “normal” individuals have a CAML genotype at *BRWD2*. Interestingly, *FGFR2* and *BRWD2*, both genes included in the GBM signature, are an oncogene and tumor suppressor pair located at a recombination locus in which deletion of exon 21 of *FGFR2* results in the exclusion of *BRWD2* (the tumor suppressor) and amplification of *FGFR2*; thus intronic CAMLs harbored by these genes could be biological indicators for tumorigenic activity. Additionally, loss of 10p is found in 47% of GBMs while 10q loss is found in 70% of primary and 63% of secondary GBMs[20]. In our GBM signature, we identified 4 loci in Ch10 at *FGFR2, BRWD2* (*WDR11*), *GLUD1*, and *NRP1;* none were identified in the LGG signature.

In the original account of ‘the two-hit hypotheses’ for somatic retinoblastoma, the first hit was an inherited mutation in *Rb*, rendering individuals more susceptible to the disease later in life. Our data suggests the ‘first hit’ may also be somatic microsatellite instability which then increases the sensitivity to, and probability of, greater genomic aberrance *theoretically* by stress factors such as environment, inflammation or age leading to the ‘second hit’[22-24]. These tentatively ‘predisposed’ variants may highlight a connection between cancer-associated microsatellites and developmental and cell cycle genes notably mutated in cancer (such as those identified in our study: *SRC, NPAT* and *CBL*); thus, these MST variants may be additive to gene mutations (including, SNPs) that contribute to cancer.

More recently, mutable genes commonly identified in GBM patients post chemotherapy and radiation treatment were discovered, and included *RYR1* [25]. *RYR1* expression is notably down-regulated in GBM; our GBM data demonstrates CAML genotypes unique to *RYR1* with transcripts and isoforms also unique to GBM. Thus, CAML loci at genes such as *RYR1* may render them to be more susceptible to mutation (for example from stresses associated with treatment) and may be valuable indicators for potential treatment outcomes. Additionally, this further supports the possibility of tumor initiating cells with irregular genetic variations that generate disease relative to an underlying combination and abundance of affected microsatellite loci.

The aberrant alteration of six helicase genes in GBM suggests that genes important to microsatellite identification and correction, along with transcription and RNA synthesis are themselves modified with MST variants. Previous studies have demonstrated that in addition to mis-match repair genes, helicases (notably RecQ family DNA helicases [26]) are important in DNA damage repair and DNA/RNA metabolism[27]. In our data we observed minimal MMR gene coding SNP mutations in both GBM and LGG tumors (see, Fig 3B) but found CAMLs significantly associated with helicases, suggesting modifications to these genes may also be important to genomic instability in glioma or that there may be more MMR genes that remain to be discovered. As such, one mechanism may be that GBM tumors produce atypical RNA. This idea is further supported by the enrichment of MST variant loci in helicase genes activated through interferon; interferon can initiate helicases and ubiquitin ligases to degrade viral RNAs and other dsRNAs (see Table S3), a process which could be interrupted if these helicases are affected by MST. As described in Table 2, we identified multiple genes in which a novel RNA isoform was the abundant transcript for several GBM genes with CAMLs, including *DICER1* and *POLQ*, both genes associated with RNA and DNA modification and metabolism.

With the abundance of helicase and UPS associated genes in our glioma signatures, another cancer promoting scenario may be introduced through changes to gene-products that compose spliceosome complexes (snRNA, snRNP, or snoRNP); through these genetic modifications, alternatively spliced RNAs may support spliceosome-associated proteins differently, which may further modify mature RNAs. A third mechanism may be modifications to ubiquitin proteasome system proteins (ligases and ubiquitin complex proteins) which could alter protein degradation or signal transduction. Supporting these two hypotheses we identified functionally associated genes with CAMLS, including: *DDX60* and *TRIM25*; *SPOPL* and *BRMS1L*; *FGFR2, BRWD2* and *CBL*; *SEMA3E* and *NRP1*. Other functional associations important to changes in RNA transcription included, *SLC44A4* transcript isoforms uniquely identified in GBM (and with expression significantly greater than normal tissue). Similar results were observed for *LNX2* in LGG supporting our hypothesis that CAMLs may modify RNA transcription and expression.

Additional important biological changes observed and linked to CAMLs included significantly decreased expression of *SEMA3E* (a GBM signature locus) compared with normal samples; as previously noted *SEMA3* competes with *VEGF*-165 for *NRP1* (a GBM signature locus) and suppresses angiogenesis [28]. Therefore, decreased expression of SEMAs in GBM suggests that these microsatellite variants could contribute to glioma angiogenesis. Several genes, including the *EVC* transcript were identified in GBM and LGG but not in normal brain tissue, and in GBM the novel isoform was the common transcript; expression data suggests that *EVC* is expressed more in GBM and LGG tumors when compared with normal germline expression, although these data did not pass our stringent 2-fold difference in expression (GBM = 1.68; LGG = 1.97).

Since these loci can be identified from germline (non-tumor) DNA, it further suggests that there is a tumor initiating cell population in which developmental cell signaling and patterning pathways may result in aberrant differentiation and proliferation due to an underlying transcriptional landscape determined by cancer-associated MST variants. Therefore, we further hypothesize that DNA microsatellite variability underlies an adaptability that is conserved in all cancers and can be investigated by determining the frequency of microsatellite variability in those genes (1) that are *essential* for cancer cell survival (and conserved across a cancer type) (2) contribute *intermittently* – to cancer cell phenotypes like metastasis, heterogeneity, or aggressiveness, and (3) are *tissue-specific*, associated with only one type of tumor or tissue origin. If so, we can predict that specific microsatellite instability contributes to cancer-specific genomics and may occur during embryogenesis, which has also been predicted in other MST associated diseases, including Huntington's disease and Fragile X syndrome[13]. Thus, these markers may be valuable to screen risk of occurrence or for treatment decisions in newly diagnosed cancer patients.

## MATERIALS & METHODS

### Microsatellite genotyping

Exome sequencing data, from Illumina HiSeq sequencing machines were obtained from The Cancer Genome Atlas (TCGA) and the 1000 Genomes Project (1kGP). These sequences were aligned to the human reference genome using BWA, by their respective projects, and re-alignment followed with microsatellite loci and variant identification by methods and software established in our laboratory [29-31]. Only loci with sequencing reads with 15x or greater depth of coverage were used to identify possible informative loci. A distribution of alleles for the affected (TCGA) and unaffected (1kGP) cohorts was then generated for each locus. An allele is defined by a genomic locus with a specific microsatellite repeat and nucleotide sequence length, in each sample a pair of alleles was identified and each allelic pair was then defined as a genotype. The genotype most prevalent from a distribution of genotypes was identified in 1kGP samples; this genotype was defined as the consensus sequence or predominant genotype (if more than a pair of alleles was identified for a locus that locus in the particular sample was not used). Similar to the 1kGP samples, LGG and GBM samples were analyzed for genotypes from the same genomic loci, loci significantly different from the consensus (predominant genotype) in one population compared with the second population were identified. The statistically significant genotypes were determined from data adjusted for false discovery rate (FDR), using a two-sided Fisher's p- test and Benjamini-Hochberg correction; relative risk (RR) was calculated for each locus and loci with a P≤ 0.01 were considered significant. More specifically, an R script computed the p-value for each locus using the two sided fisher-test function. The Benjamini-Hochberg cut-off was selected as approximately 0.01% (computed as the FDR < 1/X (where x is the total number of loci with p-value < 1 for the signature)) to make it unlikely that any locus is a false positive from our data set. Those genotypes, although individually significant and informative, were also assembled into a ‘signature’ or ‘cancer-associated’ informative loci set which together increase the statistical significance across all samples. Sequences included 390 (n=249 female; n=141 male) normal/healthy samples from the 1kGP, GBM germline (n=252), GBM tumor (n=139), LGG germline (n=136), and LGG tumor (n=158) sequencing samples (dbGAP Study Accession: phs000178.v8.p7) [32] through the Cancer Genomics Hub (CGHub). Microsatellite genotypes were identified through a software system developed in our laboratory [30, 31]. These samples, like all others, were processed to remove any reads that did not meet the QC thresholds required in the 1kGP [33]

### Creation of microsatellite target set

We produced a set of over 850,000 microsatellite loci which have flanking sequences unique in the human genome. Initially a set of over a million microsatellites was found in the human genome (NCBI36/hg18) using Tandem Repeats Finder (TRF) [34], with previously established parameters. A series of filters are in place to remove tandem repeat sequences previously identified through RepeatMasker in the flanking sequences of our loci, microsatellites shorter than 12 nucleotides in regions other than exons for which we allowed a minimum length of 10 nucleotides, those containing SNPs, indels, or SNP variations that account for variability greater than 10% of the locus lengths (Repeat Masker Ref. Smit AFA; HR Green). A custom Perl script was written to filter repeat sequences lacking unique flanking regions (10 base flanking sequences were chosen because Illumina reads are 100 bases in length, respectively); filtered sequences were screened based on corresponding flanking sequences to the reference genome (microsatellites with flanking sequences within 200 bases of each other and 5 bases of the repeat in between were also excluded). Vetted microsatellite loci are described with genomic location and gene associations using RefSeq data obtained through the UCSC Genome Table Browser [35].

### Identifying repeat lengths using microsatellite-based genotyping

Reads are mapped to the reference genome using BWA or BWA-sw (for long reads; LS454) [36]. A custom Perl script with SAMTOOLS [37] filters for reads with MST loci, to assure the span of the MST sequence is captured, a 5 base flanking sequence on either side of the read are tested, this process is to validate BWA aligned sequences. Reads with identical repeat sequences are binned; separating reads with different SNPs. SNPs contained in microsatellites were ignored for this study.

Accuracy estimations of our microsatellite-based genotyping method: Using Sanger sequencing and data from HapMap, we were able to validate 96.5% of a subset of 85 non-synonymous variations composed of repeat length variations and SNPs contained in microsatellites. The novel variants we validated using Sanger sequencing were submitted under the lab handle SGARNER and are available on-line in the latest release of dbSNP. All microsatellite-based genotyping calls from these studies were made publically available at MicrosatDB (http://discovery.vbi.vt.edu/MicrosatDB/) [31].

### Microsatellite identification restrictions for population-based statistics

To increase uniformity of coverage and genotyping rates across samples sequenced at different times with different methods by different studies, we required at least 15,000 microsatellite loci to be called per sample for inclusion in this study. This filtered out one 1kGP-F sample and 235 1kGP-M samples. From the TCGA samples, this filtered out 23 LGG germline samples, 57 LGG tumor samples, 238 GBM germline samples, and 254 GBM tumor samples. The large number of GBM samples filtered is expected as these samples were sequenced much earlier by TCGA than the LGG samples. So we would expect lower coverage and shorter reads. Only those loci with at least 15x coverage are considered “callable” in a given sample (healthy or cancer genomes). A locus had to be called in a minimum of 10 exomes to be included in the genotype distribution comparison analysis and to remove loci which may be called at insufficient frequency in one of the two data sets.

### Alternative splicing

We processed 339 GBM tumors, 268 LGG tumors, and 57 LGG germline RNAseq Illumina data sets with Cufflinks [38] by using the CuffCompare function to identify possibly alternatively spliced transcripts. These samples had been aligned by TCGA to the reference using TopHat[39]. For each transcript for each sample, we determined it was possibly alternatively spliced if one of the transcripts called by CuffCompare was not a complete match of the intron chain. Additionally, RNAseq data was downloaded for two normal brain tissue samples, adult [16]. These RNAseq SOLiD fastq read files where reformatted into standard color-space paired files and then run through TopHat using the default parameters, except for indicating the color-space type, as was done by the TCGA to align the GBM/LGG samples. These samples were then processed using Cufflinks to identify possibly alternatively spliced transcripts.

### CpG Island and TFBS Analysis

Locations of CpG islands and transcription factor binding sites associated with the human reference genome were downloaded from the UCSC Genome Browser [35]. A Perl script was written to determine the distance of each microsatellite loci from the nearest CpG island and transcription factor binding site. Methylation data was downloaded for the samples from TCGA. This data provided the CpG islands and corresponding gene with respect to each methylation site. A Perl script was written to compute methylation averages for each site for samples with and without CAML signature loci for the corresponding gene.

### Identification of Driver Mutations in GBM & LGG Samples

Variant calls for the GBM and LGG samples were downloaded from TCGA. The driver mutations and MMR genes of interest (IDH1, PTEN, PIK3CA, RB1, MSH2, MSH3, MSH4, MSH5, MSF6, MLH1, and MLH2) were analyzed with respect to the CAML status of the samples.

## SUPPLEMENTARY FIGURES AND TABLES


