# Supplementary material for: Somatic intronic microsatellite loci differentiate glioblastoma from lower-grade gliomas
Source: Oncotarget. 2014 Jun 5;5(15):6003–14. doi: 10.18632/oncotarget.2076 (PMC4171608; doi:10.18632/oncotarget.2076)
Supplement: Supplementary file 1 [file oncotarget-05-6003-s001.pdf]

Somatic intronic microsatellite loci differentiate glioblastoma from lower-grade gliomas

1. Specificity / Sensitivity and ROC plot

(1) LGG vs. 1kGP

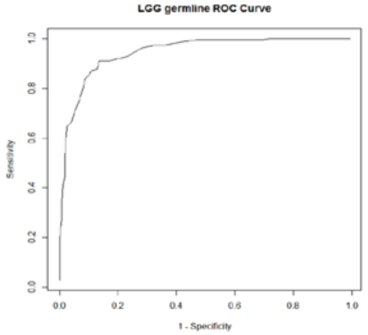

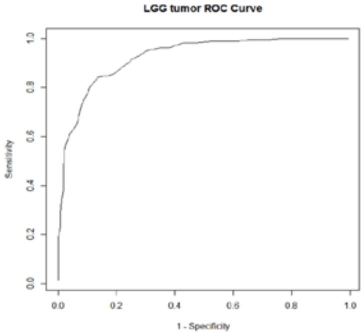

> AUC(roc.LGGgerm)

[1] 0.9340392

> AUC(roc.LGGtumor)

[1] 0.9122038

> uniqSS(roc.LGGgerm, cutoff.vec)

use cutoff: 35

sensitivity.LGGgerm specificity.LGG

0.9101124 0.8641026

> uniqSS(roc.LGGtumor, cutoff.vec)

use cutoff: 35

sensitivity.LGGtumor specificity.LGG

0.8417722 0.8641026

(2) GBM vs. 1kGP

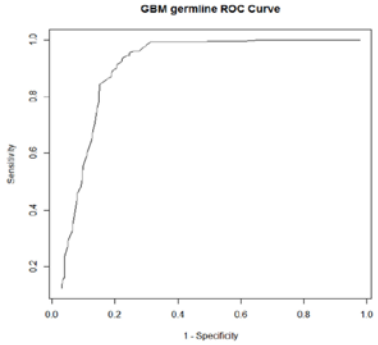

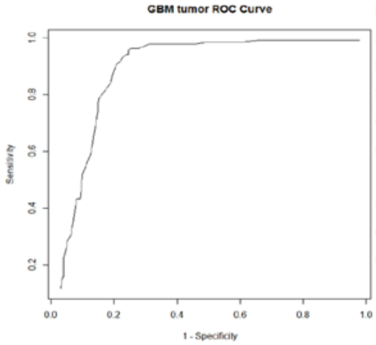

> AUC(roc.GMgerm)

[1] 0.8648555

> AUC(roc.GMtumor)

[1] 0.8521491

> uniqSS(roc.GMgerm, cutoff.vec)

use cutoff: 57

sensitivity.GMgerm specificity.GM

0.9365079 0.7743590

> uniqSS(roc.GMtumor, cutoff.vec)

use cutoff: 54

sensitivity.GMtumor specificity.GM

0.9568345 0.7538462

(3) GBM vs. LGG

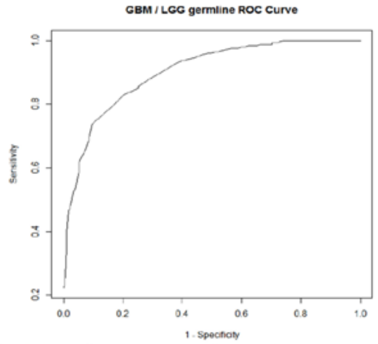

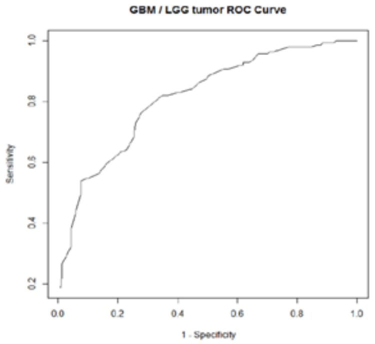

> AUC(roc.germ)

[1] 0.8946406

> AUC(roc.tumor)

[1] 0.8020249

> uniqSS(roc.germ, cutoff.vec)

use cutoff: 82

sensitivity.germ specificity.germ

0.7380952 0.9044944

> uniqSS(roc.tumor, cutoff.vec)

use cutoff: 75

sensitivity.tumor specificity.tumor

0.7625899 0.7215886

(4) GBM germline vs. LGG germline grade 2

> AUC(roc.GMLGGg2)

[1] 0.6795334

> uniqSS(roc.GMLGGg2, cutoff.vec)

use cutoff: 85

sensitivity.GMLGGg2 specificity.GMLGGg2

0.9007937 0.6969697

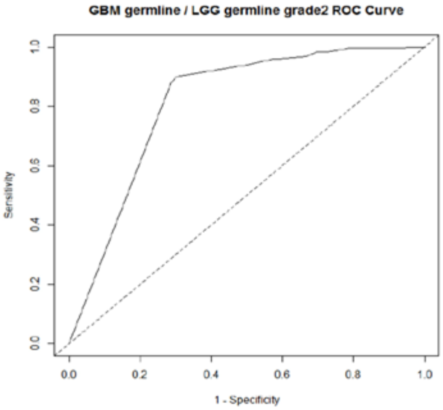

Gene	Loci Near CpG Islands (≤10 kB)	Gene	Loci within TFBS (± 40 B)	Gene	Gene Expression GBM/Normal
**Glioblastoma Multiforme (GBM)**					
RYR1	8967	9:42626-42640	CDP<br>GATA	SEMA3E	0.22
21:10017859-10017871	4575	FRMD7	HOX13	SLC44A4	2.05
LAMP1	10983	ARL13B	HMX1		
GTPBP8	9525	GTPBP8	CETS1P54		
TTF2	1861	ENAH	POU3F2<br>OCT1		
PSME3*	533	EVC*	STAT5A		
NSUN5	587	ATG3*	POU3F2<br>FOXJ2		
SLC44A4*	1409	BRMS1L	FREAC4		
TRIM25	9245	NSUN5	TAX<br>CREB<br>PAX4<br>NGFIC<br>STAT5A		
DDX20*	6415				
**Lower Grade Gliomas (LGG)**					
CBS	2291	KLRAQ1	AML1<br>EN1<br>MSX1	LNX2	0.38
RAB2B	8139	DEC1	IRF7	FGD6	0.39
CDH16	9017	ATM	SEF1.C	CRISP1	2.20
CDC16	1224	UBXN7	GFI1		
CHODL	10936	15:41789963-41789991	YY1		
HTRA4	6950	GPR125	BACH1		
		ACOXL	E4BP4<br>HLF		
		C4orf32	OCT1		
		SLC25A13	NCX		
		REL	POU3F2		
		MYCBP2	NF-kB<br>NF-kB<br>SOX5		
		KLHL3	FREAC4		
		TLN2	MEIS1<br>CDPCR3HD<br>PBX1		
		POLR3GL	OCT1<br>E4BP4<br>HLF<br>CREBP1		
		9:42626-42640	CDP<br>GATA1		
		SLC25A13	EVI1<br>PAX2		
		RBM5	CEBP<br>YY1		
		C1orf77	CEBPB<br>CEBP		
		C8orf38	MIF1<br>PPARγ		
		INTU	OCT1<br>POU3F2		
		KIF1B	YY1		

FIGURE S1: **Sensitivity & Specificity receiver operator characteristics (ROC) of Signature Cancer-Associated Microsatellite Loci in Glioma Germline and Tumor Samples.**

Table S1: **Compilation of Significant Genomic Features near Microsatellite Variants & Gene Expression Analyses:** Genes with the '*' were also identified with MST variant loci in LGG. The location of CpG islands was determined up-to 20kB, those greater than 20kB are described accordingly. Listed are those CAMLs within 10kB of CpG islands along. Transcription factor binding sites (TFBS), using locations identified in the UCSC genome browser and ENCODE, are associated with loci which are within 40 bases from these sites. CAMLs near TFBS are listed. Gene expression data from GBM and LGG tumor samples were compared with normal germline sequences from the 1kGP. Here we describe the ratio of tumor versus normal germline expression, a two-fold difference was considered significant.

GBM Gene Expression					
Genes with Cancer Associated Loci	Cancer (C) Associated Loci		Non-Cancer (NC) Loci		Ratio
	Avg.	St.Dev.	Avg.	St.Dev.	C / NC
SEMA3E	9.47	12.06	42.4	22.38	0.22*
RYR1	1.19	0.14	2.01	0.66	0.59
COL9A1	9.86	5.55	16.01	NA	0.62
BRMS1L	1	0.33	1.37	0.56	0.73
TNIK	4.14	2.88	5.57	0.49	0.74
OXR1	1.65	0.46	2.18	NA	0.76
TTF2	0.44	0.07	0.51	0.17	0.86
ICA1L	2.82	1	3.13	2.03	0.90
CBL	1.09	0.27	1.18	0.42	0.92
CORIN	1.1	0.32	1.12	0.18	0.98
ENAH	1.66	0.42	1.69	0.62	0.98
GTPBP8	1.22	0.43	1.18	NA	1.03
ATG3	0.98	0.29	0.93	0.25	1.05
NRP1	1.63	0.66	1.52	0.51	1.07
DHX36	0.77	0.18	0.71	0.2	1.08
ARL13B	1.93	0.74	1.77	0.82	1.09
NUFIP1	1.23	0.09	1.11	0.38	1.11
KCTD20	0.7	0.27	0.63	0.33	1.11
FRMD7	1.36	0.65	1.2	0.23	1.13
FUBP3	0.91	0.36	0.8	0.2	1.14
OFD1	0.72	0.24	0.62	0.09	1.16
LAMP1	0.91	0.42	0.78	0.56	1.17
PSME3	1.18	0.26	1.01	0.2	1.17
TRIML1	0.82	0.32	0.68	NA	1.21
ACRC	1.32	0.69	1.07	NA	1.23
STRC	1.04	0.39	0.81	0.36	1.28
NBPF1	1.47	0.75	1.12	0.84	1.31
DDX20	1	0.23	0.76	0.04	1.32
POLQ	0.2	0.1	0.14	NA	1.43
DPY19L2P2	0.82	0.55	0.54	0.26	1.52
BRWD2	1.18	0.45	0.73	0.23	1.62
EVC	0.94	0.49	0.56	0.33	1.68
SLC44A4	0.39	0.17	0.19	0.04	2.05*

LGG Gene Expression					
Genes with Cancer Associated Loci	Cancer (C) Associated Loci		Non-Cancer (NC) Loci		Ratio
	Avg.	St.Dev.	Avg.	St.Dev.	C/NC
LNX2	0.22	NA	0.56	0.02	0.38*
FGD6	0.51	NA	1.30	NA	0.39*
CHODL	1.67	0.85	2.66	1.05	0.63
C4orf32	0.13	NA	0.20	0.11	0.63
FNDC3B	0.27	0.06	0.38	0.15	0.70
RBM5	1.01	NA	1.24	NA	0.82
YTHDC2	0.47	NA	0.56	0.12	0.84
XAGE3	0.24	NA	0.27	0.13	0.86
PDPR	0.92	0.16	1.06	0.38	0.87
GPR125	1.83	0.33	2.11	1.15	0.87
KCTD20	0.74	0.08	0.85	NA	0.87
CSN3	0.78	0.20	0.88	NA	0.88
RAB2B	1.05	0.13	1.18	0.09	0.89
SLC44A4	0.26	0.00	0.28	NA	0.93
KLHL3	2.00	NA	2.12	1.53	0.95
NPAT	1.81	0.24	1.89	0.49	0.96
FUBP3	2.23	0.53	2.24	0.33	1.00
NCOA7	0.56	0.21	0.56	NA	1.01
ZZEF1	1.73	0.26	1.71	NA	1.01
CDH16	0.26	0.00	0.25	0.10	1.04
DIP2B	2.51	1.02	2.41	0.99	1.04
PSME3	1.21	0.21	1.07	0.16	1.13
SNX25	0.96	0.55	0.79	0.26	1.21
NCAPD3	0.55	0.40	0.44	0.12	1.27
C1orf77	0.90	0.11	0.66	0.03	1.36
LAMP1	1.70	0.93	1.23	NA	1.38
TTC13	2.06	0.37	1.21	NA	1.70
C8orf38	0.68	0.29	0.39	0.20	1.76
EVC	0.44	0.19	0.22	0.06	1.97
CRISP1	1.13	0.19	0.51	0.48	2.20*

Table S2: **Correlation of Signature LGG & GBM Microsatellite Loci with Gene Expression:** Differentially expressed genes that are significant ($p \leq 0.01$) are denoted with '*'. From signature LGG loci (blue table) two genes (*LNX2* and *FGD6*) demonstrate significantly less expression compared with non-cancer samples and increased expression of *CRISP1*, a gene whose functions are notably associated with sperm-egg fusion and is expressed in the testis. LNX2 is associated with NUMB, a membrane protein that is important to development and binds NOTCH1. *SLC44A4,* a sodium solute transporter, shows higher expression in GBM compared with non-cancer gene-expression; solute carriers have previously been linked to neuropathies and some with GBM.

Interestingly, *SLC44A4* expression is not significantly different in LGG which may suggest a biological difference in disease etiology between LGG and GBM, including the activity of shared signature loci.

Biological Process	Genes	Gene Functions
**RNA Processing**	*Helicases*<br>DHX36<br>DICER1<br>TTF2<br>DDX60<br>DDX20<br>POLQ<br>RBM5<br>SSX<br>YTHDC2<br>C1orf77<br>RRAP2<br>DEC1	Helicases with cancer-associated microsatellite loci function in splicesome complexes (*DHX36, DICER1*, and *TTF2*) and ribonucleoprotein complexes (RNPs, snRNPs, or snoRNPs) including *DDX20, DHX36*, and *DDX60*. MicroRNA synthesis, specifically affecting tumor suppressing miRNAs, is linked to multiple genes with GBM signature loci. DDX20 contributes to miRNA containing RNP complexes which suppress NF-kB via modulation of miRNA-140 (putative tumor suppressor). Epigenetic changes to mRNA and miRNA are controlled through DHX36 and DDX20. DHX36 is known to deadenylate and degrade mRNA. DNA methytransferase (*DNMT*) is regulated by miRNA-140. Where *DDX20* expression is deficient, hypermethylation at metallothionein genes by DNMT leads to decreased expression of miRNA-140 and increases NF-kB activity. miRNA are non-coding small RNAs that can regulate DNA expression post-transcriptionally; these sequences can bind to the 3' UTRs of mRNA and degrade or inhibit translation. *Thus, changes in transcription, RNA synthesis, chromatin condensation, histone/DNA/RNA methylation status and regulation of inflammation in gliomas could be accompanied by these changes.*<br><br>**Shared function by genes with CAMLS: DDX60 regulates RIG-I/MDA5 helicase complex; TRIM25 ubiquinates RIG-I; all are activated though interferon/viral RNAs.**
**Ubiquitin Proteasome System**	PSME ATG3<br>TRIM25 (EFP)<br>TRIML1<br>SPOPL CBL<br>UBXN7<br>MYCBP2<br>ATG3 KLHL3<br>C8orf38<br>NCAPD3	*MYCBP2*- a key regulator of c-Myc and proteasome degradation, *UBXN7- (*which functions with HIF1-α and transcription activators FAF2, RBX1, DLX1/6, TCEB1 and several others), *KLHL3, NCAPD3, CDC16*, and *C8orf38* in LGG). Protein modification at ubiquitin binding loci can prevent a protein's degradation, especially in the case of cancer (ref). Of these, E3 ligases with RING and Cul-3 domains were significantly represented in our signatures. *SPOPL* is a part of the E3-ubiquitin ligase complex and mediates glioma-associated oncogenes Gli2 and Gli3, both zinc-finger transcription factors downstream of sonic hedgehog signaling (Shh) (ref). Also, SPOPL, with SPOP, regulates *BRMS1L* (also a gene in the GBM signature loci) with Cul3 domains; BRMS1L is a tumor suppressor that regulates the expression of metastasis suppressing miRNAs (mi-146a and miR-146b). Another well-known E3 ubiquitin ligase, *CBL,* recognizes activated tyrosine kinases including *FGFR, PDGFR, EGFR, FLT1, KIT* and others which are over-expressed or mutated in GBM.<br><br>We found that 96% of GBM germline samples have a cancer-specific MST genotype associated with *CBL* compared to 57% of the "healthy" population. *CBL* is an ubiquitin E3 ligase; mutations in this gene are a well-known contributor to tumorigenesis. TRIM25 and TRIML1 are associated with miRNAs and RNA synthesis. *TRIM25* (also known as estrogen-responsive finger protein; *EFP*) is activated through interferon and ubiquinates DDX58 (a signature helicase described above). Additionally, TRIM25 is an RNA binding protein that is preferentially expressed in embryonic stem cells (ESCs) and is down-regulated in embryoid bodies. *TRIML1* is produced during pre-implantation in ESCs to blastocysts and is otherwise only detected in adult testis.<br><br>**Shared functions by genes with CAMLS: SPOPL functions with SPOP. SPOP regulates tumor suppressor BRMS1L; BRMS1L regulates metastasis suppressor miRNAs (146a/146b)**
**Cell Cycle & Development**	NCOR1 DIP2B<br>NEO1 FRMD7<br>KCTD20<br>FUBP3<br>BRWD2<br>CDC16 NPAT<br>RBM5 LNX2<br>CDRT KIF1B<br>KLAQ1 SNX25<br>KIF1B XAGE3	*NCOR1* is a component of a repressor complex that is recruited to methylated CpG dinucleotide islands; which are prognostic indicators for gliomas. Additionally, NCOR1 contributes to transcriptional repression by regulating nuclear receptors and promotes histone deacetylation to form repressive chromatic structures to prevent basal transcription. Cell cycle genes with cancer-associated genotypes included *CDC16* (a part of the APC complex and an E3 ubiquitin ligase that regulates G1/M phase transition) and *NPAT* (G1 to S phase transition). NPAT also positively regulates *ATM* - a transcriptional repressor that binds RB1 promoters, *MIZF* - a transcriptional activator that promotes H4 and also methylates CpG islands and *PRKDC* which promotes and activates transcription of several histones with MIZF. This suggests that NPAT could be vital to DNA damage repair and cell proliferation and therefore a potential therapeutic target. Additionally, we again see sets of genes (*ATM* and *NPAT*) with functional associations and LGG cancer-associated microsatellite genotypes. Several loci from GBM or LGG were identified with genes important to early neuronal development, progenitor cell development, and neuronal cell differentiation which are often exploited in cancer cell proliferation including: *FRMD7, FUBP3, NEO1, DIP2B, LNX2, OFD1, SRC (*which interacts with *ESR1, CBL* a signature loci, *EGFR, BCAR1, STAT3* and several other transcription regulators*), NBPF1, MYCBP2, KIF1B, KLAQ1,* and *BEND2* -BEND domains are found in proteins which function in chromatin restructuring and transcription, including alternative splicing. *FUBP3* modifies gene expression and interacts with ssDNA; similarly, mutations in *FUBP1* along with *IDH1* have previously been linked to OD
**Cell : Transport Matrix & Metabolism**	RYR1<br>TLN2<br>LAMP1<br>SLC44A4<br>GLUD1<br>NCOA7<br>SLC25A3	Calcium ion (Ca^{2+}) regulated transporters and channels were associated with GBM signature loci, including *RYR1* which mediates exocytosis of secretory vesicles and is a sensor for Ca^{2+} in the environment. Genes associated with adhesion through integrin associated pathways (*TLN2),* cell signaling and ligand presentation to selectins (*LAMP1;* has previously been implicated in cancer metastasis), transport (*SLC44A4*), and metabolism (*GLUD1)* were identified. GLUD1 contributes to glutamate being hydrolyzed into α-ketoglutarate (KG) a metabolic process that may promote gliomagenesis in cancer cells with *IDH-1* mutation. Additionally, GLUD1 is co-expressed with MDH1/2 which oxidize malate into oxaloacetate, it also increases glutamate turnover. *NCOA7* (a OXR1 family protein and OXR1 protects against oxidative damage) contributes to the transcription and co-activation of nuclear receptors, including *ESR1, THRB, PPARγ*, and *RARA*, additionally this gene is highly expressed in the brain and *OXR1* is a signature loci found in GBM; suggesting genes protective against oxidative processes may have important functions in gliomas, especially during hypoxia or increased production of reactive oxygen species (ROS) due to glioma metabolic processes. Two signature loci were identified in *SLC25A13* which is a Ca2+ dependent transporter exchanging glutamate for aspartate, as previously described glutamate metabolism can contribute to glioma phenotypes, dependent on *IDH1* mutation; this protein also interacts with BRE (brain and reproductive organs) and is a modulator of TNFRSF1A and is also a component of the BRCA1-A complex.
**Angiogenesis**	SEMA3E<br>FGFR2<br>TNIK<br>NRP1<br>CBL	We identified several other key genes associated to tyrosine kinase receptor pathways, many of which have previously been identified with cancer, including: *FGFR2, TNIK*, and *NRP1*. *SEMA3E* (contains a GBM signature locus) may down-regulate emergent angiogenesis, a balance between SEMA3s and VEGF-165 binding to KDR are regulated through *NRP1* (which also contains a GBM MST variant); therefore *NRP1* and *SEMA3E* could be therapeutic targets and loci that require further study. Supportive of this idea, *SEMA3E* RNA expression was significantly ($P \leq 0.01$) decreased in GBM tumors compared to "healthy" germline samples (Figure S2).<br><br>**Shared functions by genes with CAMLS: SEMA3E compete with VEGF-165 for KDR binding and both are regulated by NRP1; CBL regulates tyrosine kinases, including FGFR2.**
**Cell Signaling**	OFD1<br>TNIK<br>CORIN<br>ARL13B<br>EVC	Several GBM signature loci were connected with genes essential to Wnt signaling (*OFD1* and *TNIK*), Notch (*CORIN*), and Hh signaling pathways (*ARL13B* and *EVC;* ARL13B may interact with OFD1, also a GBM/LGG signature loci); these pathways are notably up-regulated in GBM and are contributive to glioma stem cell proliferation.

Table S3. **GBM/LGG Gene Ontologies and Functions.** **GBM:** GO terms over-represented (p≤0.1) in comparison to a reference *Homo sapiens* gene list are reported as identified through DAVID[3,2] annotation tools. STRING 9.1[1] and UniPROT search tools were used to identify protein-protein interactions and gene functions. From our GBM data, terms associated with key functions included helicase activity (6 genes); neurogenesis (3), alternative splicing (22), ubiquitin conjugation pathways (4), and polymorphism (29) were identified. Of these, 'helicase' was highly significant (P ≤ 0.05; 9.13×10^{-4} with Bonferroni correction). Biological processes that complemented these functions were also identified, and included: ribonucleoprotein complex assembly (3 genes), transmembrane receptor protein tyrosine kinase signaling pathway (3), autophagy (2), RNA processing (4), and proteolysis / cellular protein catabolic processes (4). Additionally, 15 genes (*STRC, CBL, LAMP1, FGFR2, ENAH, TNIK, POLQ, BRWD2, SEMA3E, PSME3, NSUN5, DICER1, NRP1, BRMS1L, SPOPL)* were identified as previously associated with cancer and three with GBM- *BRWD2* (WD repeat domain 11), *NRP1* (neuropilin1), and *FGFR2*. **LGG:** Here we analyzed a population of Grade II and III OD, OA, and A from a collective population of 178 samples, referenced as LGG. The LGG cancer-associated signature loci included 66; nine of these were also identified in the GBM signature (*PSME, LAMP1, FUBP3, ATG3, EVC, SLC44A4, NEO1* and *DDX20*) and 2 loci in intergenic regions. From 16 of the 66 loci, are linked to genes previously identified with cancer, including: *PSME, DEC1* (a tumor suppressor that deacetylates HDAC1/2- deacetylation of core histones is important to epigenetic repression and transcriptional regulation)*, ATM, LAMP1, GPR125, ACOXL, RAB2B, REL* (interacts with multiple NF-ĸB binding partners that regulate inflammation, immunity, differentiation, cell growth, tumorigenesis and apoptosis*, HAVCR2* (mediates immunotolerance)*, XAGE3, CT45-1, RBM5* (regulates alternative splicing of mRNA and is a part of the splicesome A complex)*, SSX2 (*transcription modulator*), SNX25* (may interact with KIF1B), *KIF1B* and *NPAT*. Nine genes were associated with male biology, including: *DEC1, ATM, XAGE3, CT45-1 (*may interact with multiple XAGE family proteins)*, SSX, WNK1, TTLL5* (interacts with TP53 and TP73)*, CHODL,* and *CRISP1.* C1orf77 interacts with several pre-mRNA modifying proteins; RNA polymerase II associated protein (RRAP2) and snRNA. Several genes highlighted are linked to other diseases or conditions with neurological or cognitive functions, including: *STR, ARL13B* and *OFD1* (Joubert syndrome), *NBPF1*, and ICA1L (a contributor to amyotrophic lateral sclerosis). A number of studies have highlighted a bias in gliomas in males compared to females. In this analysis, within the signature loci we observed eight genes contributive to male specific biological processes, including the following: *OFD1*, *STRC* (with exonic repeat CAG), *FRMD7*, *BRWD2*, *DICER1*, *HYDIN* (may interact with neuroblastoma breakpoint family genes 1,9,10, and 12; a duplicate copy is found on Chromosome 1), *DHX36*, *DPY19L2P2, and DDX20*. ***Disease-Associated Genes & Links to Male-Associated Biology:*** Several genes highlighted are linked to other diseases or conditions with neurological or cognitive functions, including: *STR, ARL13B* and *OFD1* (Joubert syndrome), *NBPF1*, and ICA1L (a contributor to amyotrophic lateral sclerosis). A number of studies have highlighted a bias in gliomas in males compared to females[4]. In this analysis, within the signature loci we observed eight genes contributive to male specific biological processes, including the following: *OFD1*, *STRC* (with exonic repeat CAG), *FRMD7*, *BRWD2*, *DICER1*, *HYDIN* (may interact with neuroblastoma breakpoint family genes 1,9,10, and 12; a duplicate copy is found on Chromosome 1), *DHX36*, and *DPY19L2P2*. DDX20 is well known for its regulation and suppression of steroidogenic factor 1 (SF-1) which is expressed in gonadal tissues. These genes have brain and testis specific expression, including spermatogenesis, and some with testis only expression. Microsatellite loci with genotypes specific for cancer may be important to GBM in males.

DDX60
Length of Normal Germline Sequence Genotypes: 15 15 , 16 15, 14 14 , 16 14 , 14 15 , 16 17 , **16 16***
Length of GBM Germline Sequence Genotypes: 13 13 , 15 15, 16 15 , 16 14 , 16 16

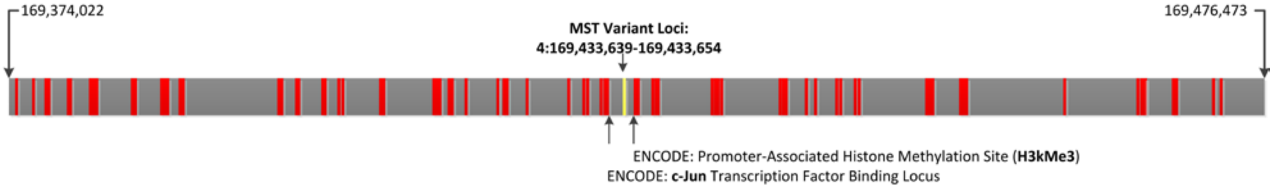

POLQ
Length of Normal Germline Sequence Genotypes: **25 24***, 25 25 , 24 24
Length of GBM Germline Sequence Genotypes: 25 24 , 24 24

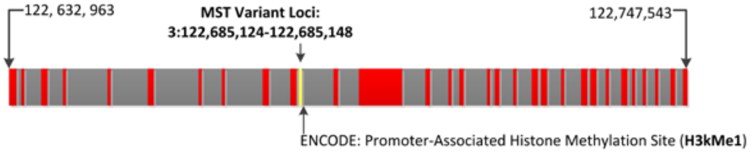

TTF2
Length of Normal Germline Sequence Genotypes: 13 14, **14 14***
Length of GBM Germline Sequence Genotypes:13 13, 14 14, 13 14

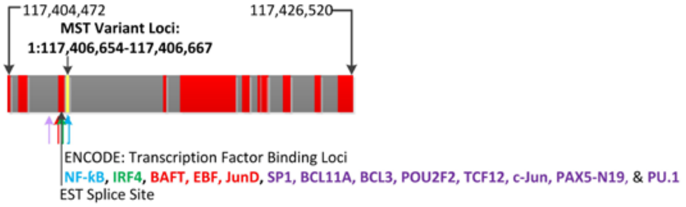

DDX20
Length of Normal Germline Sequence Genotypes: 16 16, 15 14, **16 15***
Length of GBM Germline Sequence Genotypes: 16 15, 16 17, 16 16

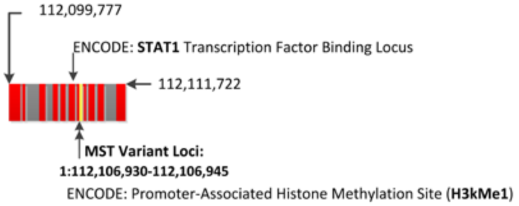

DHX36
Length of Normal Germline Sequence Genotypes: **12 12***, 13 13 , 13 12
Length of GBM Germline Sequence Genotypes: 13 13, 11 12, 13 12 , 12 12

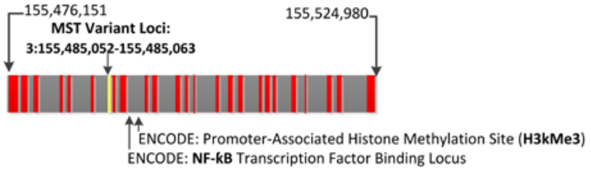

DICER1
Length of Normal Germline Sequence Genotypes: 27 37, 27 29, 39 41, 39 39, 35 37, 39 37, 27 27, 29 29, 37 29, 37 41, 27 31, 31 31, **37 37***, 29 41
Length of GBM Germline Sequence Genotypes: 37 29, 35 37, 31 31, 37 37, 27 27, 29 29

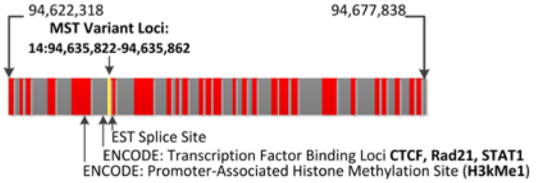

Figure S2. Described are six helicases with variant microsatellite loci identified in GBM. Helicases are important to RNA decay and remodeling. At the location of each MST variant (yellow) we have also described important genomic elements, including: histone methylation markers described through ENCODE (H3kMe3 or H3kMe1), transcription factor binding loci, and ESTs/splice sites. The total length of the gene and the microsatellite loci are identified, with exons (red). Included are the lengths of prevalent microsatellite allelic pairs (genotypes) from normal and GBM germlines, with the consensus denoted ('*****'and in blue). Variants at these microsatellite loci could change gene/exon transcription or expression due to their location near histone methylation markers, transcription factors binding loci, or splice sites.

**Glioma**	**Signature Loci**	**Significant Loci**	**Population**
Oligodendroglioma Grade II and III	SEC31B<br>TNPO1<br>PDPR<br>DIP2B<br>ATL1<br>15:41789963-41789991	81	~ 91
Astrocytoma Grade II and III	-	16	~ 50
Oligoastrocytoma Grade II and III	-	27	~64

Table S4. CAMLs and Significant MST Loci in LGG: OD, A, & OA. Sequence samples that were collectively used for the LGG signature were separated into each of three diagnostic groups and analyzed independently for significant and signature loci. CAMLs were identified for OD. A and OA populations were below the necessary cut-off to identify CAMLs, however significant loci were identified and are further described in the supplemental data in the data spreadsheet file, titled 'Glioma_ Cancer Associated Microsatellite Loci'.

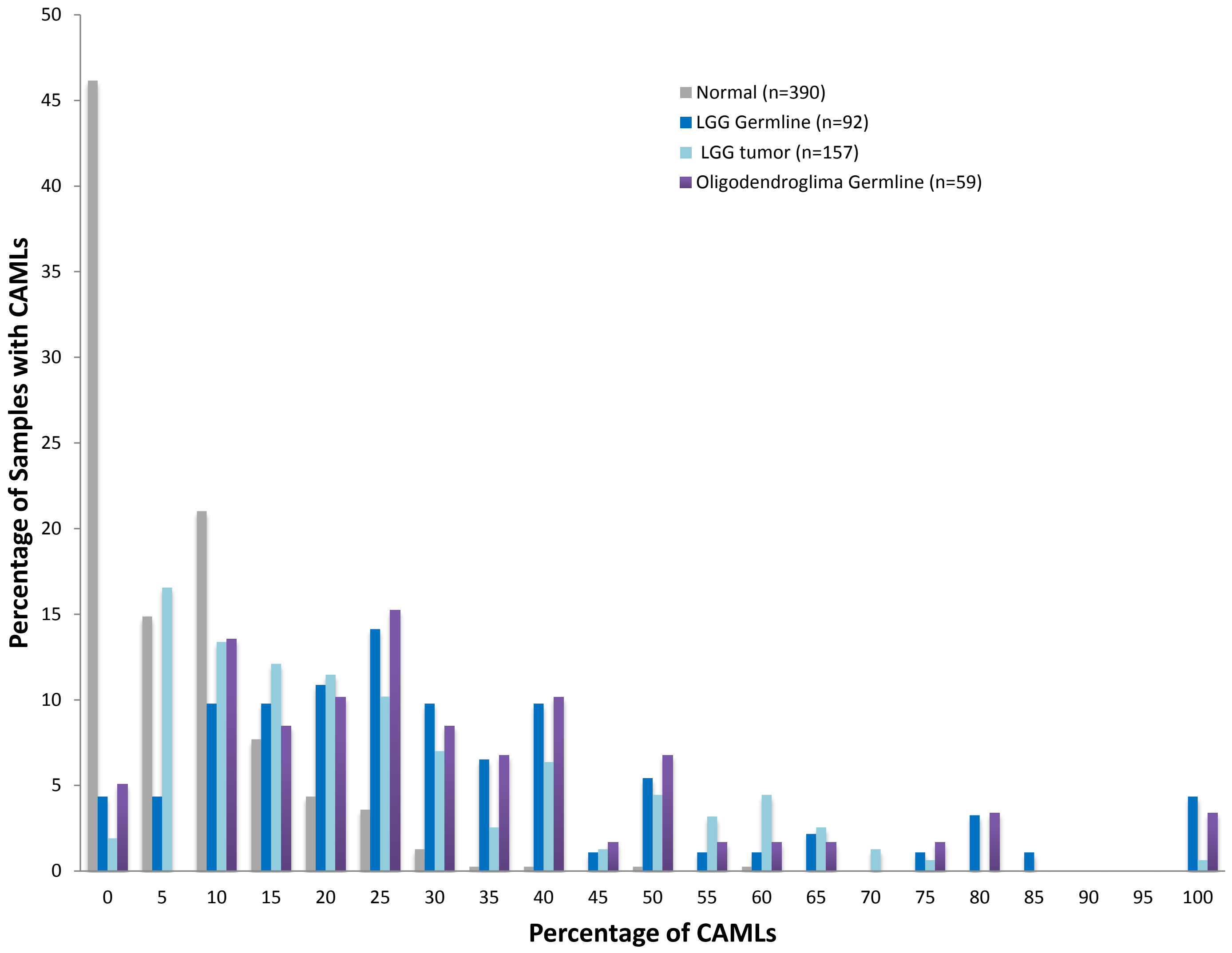

Figure S3. LGG CAMLs (without Grade III Astrocytomas): For this analysis we removed Grade III astrocytomas (n=28, additionally samples with non-descript pathology were removed, 16 =NA and 2=UNK) to create an LGG signature (sensitivity = 88%; specificity = 76%; cutoff = 13%). This LGG signature is composed of 24 CAMLs, of which all but 4 loci were in the LGG signature described in Figure 1B; however all 4 were identified as significant loci in the original analysis. This demonstrates that the original signature is mostly comprised of loci important to lower-grade gliomas. Additionally, we graphed Oligodendroglioma (Grade II and III) samples (n= 59, the largest LGG sub-population) to determine the percentage of these samples with LGG CAML genotypes. We observed that up to 15% of OD samples showed 25% of these CAML genotypes and most OD germline samples (purple bars) showed a similar percentage of CAMLs compared with the LGG germline samples (dark blue bars).

location (hg19)	FDR adjusted p-value	normal diff	normal same	cancer diff	motif	region	gene
2:91886031-91886042	3.25E-10	42	275	90	A	intergenic	NA
2:87122106-87122120	5.91E-09	18	110	37	T	intergenic	NA
19:39077896-39077911	5.98E-07	1	125	13	AT	intron	RYR1
9:52626-52640	4.74E-06	31	31	80	A	intergenic	NA
X:13775753-13775768	1.23E-05	55	46	60	T	intron	OFD1
21:10995988-10996000	2.15E-05	5	293	19	A	intergenic	NA
15:43910867-43910899	2.13E-05	5	238	22	CAG	exon	STRC
X:131231431-131231468	2.66E-05	36	51	40	AC	intron	FRMD7
3:93754287-93754302	3.22E-04	6	40	25	T	intron	ARL13B
11:119144792-119144808	3.42E-04	31	36	2	T	intron	CBL
4:169197064-169197079	3.69E-04	32	41	28	A	intron	DDX60
6:36452604-36452619	3.68E-04	39	34	1	A	intron	KCTD20
1:16890815-16890826	4.66E-04	24	82	4	A	intron	NBPF1
13:113964899-113964910	5.73E-04	28	29	69	T	intron	LAMP1
3:112719792-112719807	6.70E-04	35	38	3	A	3utrE	GTPBP8
1:117605131-117605144	1.02E-03	9	99	14	T	intron	TTF2
9:133498230-133498244	1.17E-03	22	36	61	A	intron	FUBP3
12:33578998-33579044	1.11E-03	32	25	35	CA	intron	SYT10
10:123256330-123256345	1.30E-03	12	22	23	T	intron	FGFR2
2:203680555-203680567	1.52E-03	36	111	4	A	intron	ICA1L
1:225707272-225707287	1.76E-03	6	22	24	A	intron	ENAH
4:5746907-5746928	1.69E-03	44	237	9	TTC	intron	EVC
3:170844017-170844030	1.76E-03	2	15	12	A	intron	TNIK
3:112253194-112253207	1.86E-03	31	27	71	A	intron	ATG3
3:121202434-121202458	2.02E-03	23	23	1	A	intron	POLQ
10:122648751-122648767	2.08E-03	102	109	18	TTTTG	intron	BRWD2
7:83021800-83021817	2.09E-03	32	27	4	A	intron	SEMA3E
10:88817579-88817594	2.07E-03	15	21	21	A	intron	GLUD1
17:40986455-40986486	3.16E-03	36	131	40	GA	intron	PSME3
15:85056104-85056118	3.53E-03	6	37	11	A	3utrI	FLJ40113
7:72721731-72721740	4.03E-03	0	274	4	CAA	exon	NSUN5
14:95566069-95566109	4.25E-03	45	52	21	AC	intron	DICER1
6:31832357-31832371	4.41E-03	41	59	34	A	intron	SLC44A4
16:70873867-70873881	4.63E-03	6	6	37	T	intron	HYDIN
6:70950282-70950298	4.60E-03	103	63	3	AT	intron	COL9A1
X:70812449-70812463	4.66E-03	18	118	0	T	intron	ACRC
17:54981572-54981587	5.51E-03	36	51	5	A	intron	TRIM25
4:189063362-189063397	5.77E-03	50	40	24	GT	intron	TRIML1
11:89502008-89502035	5.87E-03	30	76	16	GA	intergenic	NA
4:47746603-47746615	6.61E-03	31	136	3	A	intron	CORIN
10:33471762-33471790	6.95E-03	22	69	30	CA	intron	NRP1
13:45517483-45517512	7.86E-03	20	235	16	AC	intron	NUFIP1
14:36334906-36334920	7.71E-03	2	38	19	T	intron	BRMS1L
1:112305407-112305422	8.44E-03	25	29	5	A	intron	DDX20
8:107704941-107704954	9.03E-03	21	154	1	A	intron	OXR1
3:154002358-154002369	9.06E-03	92	122	38	T	intron	DHX36
7:102825988-102826000	9.83E-03	32	190	8	A	3utrI	DPY19L2P2
2:139308384-139308419	9.76E-03	46	30	27	TC	intron	SPOPL

GBM Signature Loci

Table S5 Signature Microsatellite Loci Identified GBM with Location and Significance.

location (hg19)	adjusted p-value	region	symbol
10:102265052-102265096	1.16E-12	intron	SEC31B
2:48688259-48688272	3.01E-06	intron	KLRAQ1
17:40986455-40986486	7.65E-06	intron	PSME3
15:73418742-73418755	5.27E-04	intron	NEO1
16:70176322-70176335	5.44E-04	intron	PDPR
9:118164376-118164387	5.07E-04	intron	1-Dec
5:72185592-72185606	4.85E-04	intron	TNPO1
3:196088810-196088825	5.82E-04	intron	UBXN7
1:145456733-145456746	1.21E-03	intron	POLR3GL
4:22444252-22444266	1.11E-03	intron	GPR125
14:21936763-21936775	1.12E-03	intron	RAB2B
14:51062237-51062261	1.30E-03	intron	ATL1
17:15973418-15973434	1.61E-03	intron	NCOR1
13:28133957-28133971	1.88E-03	intron	LNX2
3:112253194-112253207	2.04E-03	intron	ATG3
7:95775849-95775862	3.15E-03	intron	SLC25A13
12:51053874-51053888	3.23E-03	intron	DIP2B
6:36452604-36452619	3.52E-03	intron	KCTD20
19:21558016-21558032	3.59E-03	intergenic	NA
15:44002671-44002699	4.05E-03	intergenic	NA
5:137013351-137013364	4.10E-03	intron	KLHL3
4:5746907-5746928	4.13E-03	intron	EVC
13:77792100-77792112	4.10E-03	intron	MYCBP2
21:44488756-44488769	4.14E-03	intron	CBS
5:94903576-94903588	4.40E-03	intron	ARSK
12:95488340-95488353	4.58E-03	intron	FGD6
3:132166149-132166161	4.55E-03	intron	DNAJC13
13:115002098-115002110	4.67E-03	intron	CDC16
X:18183098-18183112	4.65E-03	3utrE	BEND2
7:65426055-65426068	4.59E-03	intron	GUSB
4:128621145-128621157	4.88E-03	intron	INTU
1:10357207-10357223	5.15E-03	intron	KIF1B
4:113107830-113107844	5.24E-03	intron	C4orf32
17:15517061-15517072	5.24E-03	intron	CDRT1
2:111721143-111721181	5.74E-03	intron	ACOXL
3:158407931-158407944	6.13E-03	intron	GFM1
13:113964899-113964910	7.90E-03	intron	LAMP1
12:21791411-21791425	8.55E-03	intron	LDHB
9:133498230-133498244	9.09E-03	intron	FUBP3
16:66946895-66946926	8.87E-03	intron	CDH16
3:50155884-50155909	8.96E-03	3utrE	RBM5
8:142161667-142161680	9.45E-03	intron	DENND3

LGG Signature Loci

Table S6 Signature Microsatellite Loci Identified in *Lower* Grade Gliomas with Location and Significance.

location (hg19)	FDR adjusted p-value	motif	region	gene
9:52626-52640	1.12E-03	A	intergenic	NA
3:45776876-45776888	3.14E-03	T	intron	SACM1L
3:158407931-158407944	2.62E-03	T	intron	GFM1
13:77792100-77792112	2.75E-03	A	intron	MYCBP2
20:37146132-37146145	3.76E-03	T	intron	KIAA1219
2:27597191-27597203	3.15E-03	T	intron	SNX17
4:83970298-83970311	2.92E-03	T	intron	COPS4
13:115002098-115002110	5.45E-03	T	intron	CDC16

Table S7 GBM vs. LGG Grade II Signature Microsatellite Loci with Location and Significance.

location (hg19)	adjusted p-value	consensus	LGG diff	LGG same	GBM diff	GBM same	motif	region	symbol
11:116691512-116691528	3.81E-13	13 17	47	149	94	42	GACA	3utrE	APOA4
6:42611937-42611950	4.12E-05	14 14	0	129	16	62	A	intron	UBR2
X:52734297-52734310	3.61E-05	14 14	1	110	10	15	A	intron	SSX2
9:52626-52640	3.48E-05	14 15	14	73	17	5	A	intergenic	NA
16:70176322-70176335	7.30E-05	13 14	21	67	28	10	T	intron	PDPR
1:148888277-148888289	5.07E-04	13 12	34	43	27	2	A	intergenic	NA
16:7703786-7703806	5.05E-04	23 23	102	82	92	20	CT	intron	A2BP1
4:128621145-128621157	4.77E-04	13 13	1	213	13	90	T	intron	INTU
2:91886031-91886042	5.29E-04	10 12	90	126	23	107	A	intergenic	NA
13:77792100-77792112	8.03E-04	13 13	7	179	18	61	A	intron	MYCBP2
13:115002098-115002110	1.03E-03	13 12	7	53	19	15	T	intron	CDC16
20:37146132-37146145	9.49E-04	14 14	0	185	11	91	T	intron	KIAA1219
3:45776876-45776888	1.22E-03	13 13	10	113	27	55	T	intron	SACM1L
11:16117685-16117697	1.45E-03	13 13	3	107	14	40	A	intron	SOX6
4:22444252-22444266	1.69E-03	15 14	20	83	14	6	A	intron	GPR125
1:181714467-181714480	1.95E-03	14 14	3	100	18	57	T	intron	CACNA1E
14:51062237-51062261	4.27E-03	25 23	54	112	35	19	TC	intron	ATL1
2:27597191-27597203	4.56E-03	13 13	0	196	9	95	T	intron	SNX17
15:73418742-73418755	4.42E-03	14 14	3	151	15	79	T	intron	NEO1
15:20666398-20666410	4.37E-03	13 13	16	130	21	35	A	intergenic	NA
11:89663425-89663438	4.80E-03	14 14	2	165	13	85	T	intron	LOC729384
3:158407931-158407944	5.01E-03	14 14	5	70	17	31	T	intron	GFM1
9:118164376-118164387	5.29E-03	12 12	4	115	15	53	T	intron	1-Dec
3:132363753-132363764	6.85E-03	12 12	2	222	8	55	A	intron	ACAD11
15:44002671-44002699	7.08E-03	29 29	4	162	13	65	TG	intergenic	NA
1:151384053-151384066	7.81E-03	14 14	0	174	6	48	A	intron	POGZ
X:23693085-23693097	7.61E-03	13 13	2	153	10	57	T	intron	PRDX4
5:137013351-137013364	7.49E-03	14 14	15	87	19	22	A	intron	KLHL3
X:52782247-52782260	7.71E-03	14 14	4	108	6	9	T	intron	SSX2

LGG vs. GBM

Table S8 LGG vs. GBM Signature Microsatellite Loci with Location and Significance

REFERENCES:

1. Franceschini A, Szklarczyk D, Frankild S, Kuhn M, Simonovic M, Roth A, Lin J, Minguez P, Bork P, von Mering C, Jensen LJ (2013) STRING v9.1: protein-protein interaction networks, with increased coverage and integration. Nucleic acids research 41 (Database issue):29
2. Huang da W, Sherman BT, Lempicki RA (2009) Bioinformatics enrichment tools: paths toward the comprehensive functional analysis of large gene lists. Nucleic acids research 37 (1):1-13
3. Huang da W, Sherman BT, Lempicki RA (2009) Systematic and integrative analysis of large gene lists using DAVID bioinformatics resources. Nature protocols 4 (1):44-57
4. Pokhrel KP, Vovoras D, Tsokos CP (2012) Histological and Demographic Characteristics of the Distribution of Brain and Central Nervous System Tumors' Sizes: Results from SEER Registries Using Statistical Methods. Int J Biomed Sci 8 (3):152-162
